# The Role of Visual Experience in Individual Differences of Brain Connectivity

**DOI:** 10.1523/JNEUROSCI.1700-21.2022

**Published:** 2022-06-22

**Authors:** Sriparna Sen, Nanak Nihal Khalsa, Ningcong Tong, Smadar Ovadia-Caro, Xiaoying Wang, Yanchao Bi, Ella Striem-Amit

**Affiliations:** ^1^Department of Neuroscience, Georgetown University Medical Center, Washington, DC 20057; ^2^Department of Psychology, Harvard University, Cambridge, MA 02138; ^3^Department of Cognitive Sciences, University of Haifa, Haifa 3498838, Israel; ^4^State Key Laboratory of Cognitive Neuroscience and Learning, Beijing Normal University, Beijing 100875, China; ^5^IDG/McGovern Institute for Brain Research, Beijing Normal University, Beijing 100875, China; ^6^Chinese Institute for Brain Research, Beijing 102206, China

**Keywords:** brain plasticity, development, individual differences, vision

## Abstract

Visual cortex organization is highly consistent across individuals. But to what degree does this consistency depend on life experience, in particular sensory experience? In this study, we asked whether visual cortex reorganization in congenital blindness results in connectivity patterns that are particularly variable across individuals, focusing on resting-state functional connectivity (RSFC) patterns from the primary visual cortex. We show that the absence of shared visual experience results in more variable RSFC patterns across blind individuals than sighted controls. Increased variability is specifically found in areas that show a group difference between the blind and sighted in their RSFC. These findings reveal a relationship between brain plasticity and individual variability; reorganization manifests variably across individuals. We further investigated the different patterns of reorganization in the blind, showing that the connectivity to frontal regions, proposed to have a role in the reorganization of the visual cortex of the blind toward higher cognitive roles, is highly variable. Further, we link some of the variability in visual-to-frontal connectivity to another environmental factor—duration of formal education. Together, these findings show a role of postnatal sensory and socioeconomic experience in imposing consistency on brain organization. By revealing the idiosyncratic nature of neural reorganization, these findings highlight the importance of considering individual differences in fitting sensory aids and restoration approaches for vision loss.

**SIGNIFICANCE STATEMENT** The typical visual system is highly consistent across individuals. What are the origins of this consistency? Comparing the consistency of visual cortex connectivity between people born blind and sighted people, we showed that blindness results in higher variability, suggesting a key impact of postnatal individual experience on brain organization. Further, connectivity patterns that changed following blindness were particularly variable, resulting in diverse patterns of brain reorganization. Individual differences in reorganization were also directly affected by nonvisual experiences in the blind (years of formal education). Together, these findings show a role of sensory and socioeconomic experiences in creating individual differences in brain organization and endorse the use of individual profiles for rehabilitation and restoration of vision loss.

## Introduction

The visual cortex has consistent functional organization and connectivity across individuals (Dehaene and Cohen, 2007; Wandell et al., 2007; Kanwisher, 2010; Kravitz et al., 2013; Weiner and Grill-Spector, 2013); however, some meaningful inter-personal variability exists (Osher et al., 2016; Tavor et al., 2016; Feilong et al., 2018).

Brain variability informs theories of brain development and experience-dependent plasticity and also has clinical relevance. Sources of variability can be traced to species-level developmental processes, showing that variability is greater in frontoparietal association cortices evolutionarily expanded in humans (Kaas, 2006; Mueller et al., 2013). Variability also hints at the developmental temporal trajectory at the individual level as changes accumulate differently across cortical sites and ages (Gao et al., 2014; Xu et al., 2018). Furthermore, variability in connectivity and activation patterns has been linked to more than a hundred behavioral abilities (Tavor et al., 2016; reviewed in Vaidya and Gordon, 2013). In addition to the variability of the healthy adult brain, individual differences in development and aging, psychiatric illnesses, and developmental disorders (Hahamy et al., 2015; Brown, 2017; Friedman and Miyake, 2017; Foulkes and Blakemore, 2018) are intensively studied to lead to a better diagnosis and individually tailored medical interventions (Fox and Greicius, 2010; Drysdale et al., 2017).

Despite the importance of interindividual variability in determining brain development and (dys)function, the origins of neural variability remain unclear. Heritability has been shown to account for a high percentage of variance (Polk et al., 2007; Park et al., 2012b; Yang et al., 2016; Ge et al., 2017; Reineberg et al., 2020; Alvarez et al., 2021) but does not explain the full range of individual differences. One large source of variability, the effects of environmental factors such as sensory experience, remains particularly unclear. Unimodal cortices that develop fully early in life show lower variability compared with later developing networks (Mueller et al., 2013; Anderson et al., 2021), suggesting that longer developmental trajectory allows longer exposure to differential extrinsic experiences and offers higher variability in late-maturing brain regions (Mueller et al., 2013; Gratton et al., 2018). But can experience also have a stabilizing effect on brain variability in cases of shared environment and consistent experience? Is the low variability of the early cortices an inherent trait of the cortical tissue of these areas or is it because of the shared early onset sensory experience in that modality? These questions broadly address the malleability of brain organization and the variability of potential outcomes when typical experience is not provided.

We tested the role of experience on brain variability in an extreme model of experience deprivation, that is, people born completely blind. In congenital blindness, the brain is deprived of the typical visual input that shapes the visual system (Wiesel and Hubel, 1963; Röder et al., 2013; Maurer, 2017). We tested whether cross-individual variability in brain connectivity, manifested in resting-state functional connectivity (RSFC), is affected by sensory experience in a homogenous group of fully and congenitally blind adults. Although RSFC is only a correlate to functional responses and anatomic connectivity of the brain (Fox and Raichle, 2007; Honey et al., 2009; Smith et al., 2009), individual differences in connectivity appear to be temporally stable (Jovicich et al., 2016; Badhwar et al., 2020), allowing their use for addressing questions of individual variability. Three possible predictions can be formulated. If all life experiences increase individual neural differences, limited visual experience will reduce interindividual variability in blindness. However, as the statistical properties of visual environmental experience in vision are highly consistent (Simoncelli, 2003; Berkes et al., 2011), visual input may have a stabilizing effect on brain variability, leading to higher RSFC diversity in blindness where cortex organization is not constrained by shared visual experience. Mechanistically, stabilization may stem from developmental pruning of variable nonvisual projections innervating V1 (Dehay et al., 1984; Innocenti and Clarke, 1984; Innocenti et al., 1988; Kennedy et al., 1989; Rockland and Van Hoesen, 1994), enforcing a more consistent resulting connectivity profile. A third alternative is that blindness would have no effect on brain consistency, indicating strong inherited stabilization of brain individuation for the early visual cortex.

In addition to clarifying the role of experience on brain individuation, discovering meaningful individual differences in blindness could explain mixed findings of the role of the V1 in blindness. The blind visual cortex responds, on average, to a large variety of sensory and cognitive tasks (Sadato et al., 1996; Weeks et al., 2000; Amedi et al., 2003; Burton et al., 2003; Gougoux et al., 2005; Stilla et al., 2008; Bedny, 2017; Mattioni et al., 2020a) and becomes more functionally connected to frontal cortices (Liu et al., 2007; Burton et al., 2014; Deen et al., 2015; Striem-Amit et al., 2015; Abboud and Cohen, 2019), raising questions about its functional role. However, despite recent evidence for some variability of auditory-based category responses in the visual cortex in blindness (van den Hurk et al., 2017; Abboud et al., 2019; Rosenke et al., 2020; Mattioni et al., 2020b), the consistency of early visual cortex reorganization has never been explicitly examined. Last, if reorganization varies among the blind, it could allow implementing individually tailored medical and rehabilitative interventions, to address the large variability in sight restoration outcomes (Gregory and Wallace, 1963; Carlson et al., 1986; Ganesh et al., 2014).

## Materials and Methods

### Participants

Twenty-five congenitally blind individuals and 31 sighted controls participated in the study. The data were collected for two previous studies (Striem-Amit et al., 2015, 2018b), scanned at two separate sites. Cohort A included 13 congenitally blind individuals (8 female) and 18 sighted controls (Striem-Amit et al., 2015). Cohort B included 12 congenitally blind individuals (4 female) and 13 sighted controls (Striem-Amit et al., 2018b). Sighted participants had normal or corrected-to-normal vision; all participants had no history of neurologic disorder. Groups within each cohort were matched for age and education. Participants in the blind group (across cohorts) were between 22 and 63 years of age (no significant group difference for each cohort separately, *p* > 0.14, *p* > 0.99, or collapsed across cohorts *p* > 0.34). Duration of formal education was also comparable across groups (*p* > 0.45, *p* > 0.97 for each cohort separately or collapsed across cohorts *p* > 0.49). Table 1 provides detailed characteristics of the blind participants in each cohort. The Tel-Aviv Sourasky Medical Center Ethics Committee approved the experimental procedure for cohort A, and the Institutional Review Board of the Department of Psychology, Peking University, China and the Institutional Review Board of Harvard University approved the experimental procedure for cohort B. Written informed consent was obtained from each participant.

**Table 1.** Characteristics of blind participants

Participant	Cohort	Gender	Age	Cause of blindness	Light perception	Handedness	Age of blindness onset
1	A	F	29	Microphthalmia	None	Right	0
2	A	F	23	Microphthalmia, retinal detachment	None	Left	0
3	A	F	30	Retinopathy of prematurity	None	Right	0
4	A	M	37	Retinopathy of prematurity	None	Right	0
5	A	F	38	Enophthalmus	None	Left	0
6	A	M	54	Retinopathy of prematurity	None	Right	0
7	A	M	23	Microphthalmia	None	Right	0
8	A	F	34	Retinopathy of prematurity	None	Right	0
9	A	M	31	Retinopathy of prematurity	None	Right	0
10	A	F	35	Retinoblastoma	None	Right	0
11	A	F	34	Microphthalmia	None	Left	0
12	A	F	30	Leber congenital amaurosis	Faint	Ambidextrous	0
13	A	M	42	Retinopathy of prematurity	Faint	Right	0
14	B	M	36	Microphthalmia	None	Ambidextrous	0
15	B	M	22	Microphthalmia	None	Right	0
16	B	M	33	Microphthalmia; microcornea	None	Right	0
17	B	M	48	Glaucoma	None	Right	0
18	B	F	46	Glaucoma	None	Right	0
19	B	M	40	Leukoma	Faint	Right	0
20	B	F	50	Cataracts; eyeball dysplasia	Faint	Right	0
21	B	M	57	Eyeball dysplasia	None	Right	0
22	B	F	43	Glaucoma	None	Right	0
23	B	M	48	Microphthalmia; cataracts; leukoma	None	Right	0
24	B	M	63	Glaucoma; leukoma	None	Right	0
25	B	F	41	Optic nerve atrophy	Faint	Right	0

Cohort A was acquired in Israel and comprised 13 blind adults and 18 sighted controls (Striem-Amit et al., 2015). Cohort B was acquired in China and comprised 12 blind adults and 13 sighted controls (Striem-Amit et al., 2018b). F, Female; M, male.

### Functional imaging

Functional magnetic resonance imaging (fMRI) data were obtained during resting conditions, without any external stimulation or task (i.e., spontaneous blood oxygen level-dependent fluctuations) for both cohorts. During the scan, subjects lay supine in the scanner with no external stimulation or explicit task. The sighted subjects were blindfolded and had their eyes shut for the duration of the scan.

#### Cohort A

Images were acquired with a 3-T General Electric scanner with an *in vivo* eight-channel head coil. Data consisted of one functional run, containing 180 continuous whole-brain functional volumes acquired with an echoplanar imaging sequence [repetition time (TR) = 3000 ms, echo time (TE) = 30 ms, 29–46 slices, voxel size 3 × 3 × 4 mm, flip angle (FA) 90°, 182 volumes, scan duration = 9.1 min]. T1-weighted anatomic images were acquired using a 3D MPRAGE sequence (typical scan parameters were 58 slices; TR = 8.9 ms, TE = 3.5 ms, inversion time = 450 ms, FA = 13°, FOV = 256 × 256 mm, voxel size = 1 × 1 × 1 mm, matrix size = 256 × 256).

#### Cohort B

Images were acquired using a Siemens Prisma 3-T scanner with a 20-channel phase-array head coil. Data consisted of one functional run, containing 240 continuous whole-brain functional volumes that were acquired with a simultaneous multislice sequence supplied by Siemens as follows: slice planes scanned along the rectal gyrus, 64 slices, phase encoding direction from posterior to anterior; 2 mm thickness; 0.2 mm gap; multiband factor = 2, TR = 2000 ms, TE = 30 ms, FA = 90°, matrix size = 112 × 112, FOV = 224 × 224 mm, voxel size = 2 × 2 × 2 mm. T1-weighted anatomic images were acquired using a 3D MPRAGE sequence (192 slices, 1 mm thickness, TR = 2530 ms, TE = 2.98 ms, inversion time = 1100 ms, FA = 7°, FOV = 256 × 224 mm, voxel size = 0.5 × 0.5 × 1 mm, interpolated; matrix size = 512 × 448). Data of cohort B were downsampled to a resolution of 3 mm isovoxels for joint analysis with data from cohort A.

### fMRI preprocessing

Data analysis was performed using the BrainVoyager 20 software package (Brain Innovation) and custom scripts in MATLAB (MathWorks) following standard preprocessing procedures. The first two images of each scan were excluded because of non-steady-state magnetization. Preprocessing of functional scans included 3D motion correction, slice scan time correction, bandpass filtering (0.01–0.1 Hz), and regression of spurious signals from the ventricles and white matter regions (defined using the grow-region function in BrainVoyager on the individual level). Head motion did not exceed 2 mm along any given axis or include spike-like motion of >1 mm in any direction. There was no difference in head displacement between the groups and cohorts (2 × 2 ANOVA for group X cohort; group effect, *F*_(1,53)_ = 0.39, *p* = 0.53; cohort effect, *F*_(1,53)_ = 1.02, *p* = 0.32; interaction *F*_(1,53)_ = 1.26, *p* = 0.27). Data were normalized to standard Talairach space (Talairach and Tournoux, 1988). Analyses were replicated (Extended Data Fig. 1-1) using global signal regression as a preprocesing step, known to aid in overcoming motion-derived artifacts and link to behavior (Ciric et al., 2017; Li et al., 2019), but also to introduce additional artifacts (e.g., introduction of anticorrelation, distortion of group differences, and exacerbation of distance-dependent motion artifacts; Murphy et al., 2009; Anderson et al., 2011; Power et al., 2012; Saad et al., 2012; Satterthwaite et al., 2012; Gotts et al., 2013; Hahamy et al., 2014; Ciric et al., 2017). To overcome differences originating from the two datasets, scan parameters and cohorts, we applied *post hoc* standardization (*z* normalization of the data), shown to dramatically reduce site-related effects (Yan et al., 2013). An additional step to exclude site-related effects was the integration of the cohort grouping factor explicitly in the RSFC ANOVA (see below) and study effects related to group regardless of the cohort (as evident by the minimal cohort effects remaining in the analyzed data; see Fig. 2*B*).

### Seed regions of interest

The region of interest (ROI) for the primary visual cortex (V1) was defined from an independent localizer, acquired in a separate group of 14 sighted subjects (Striem-Amit et al., 2015) using a standard phase-encoded retinotopic mapping protocol, with eccentricity and polar mapping of ring and wedge stimuli, respectively (Engel et al., 1994; Sereno et al., 1995; Wandell et al., 2007; Wandell and Winawer, 2011). The experimental detail can be found in Striem-Amit et al. (2015). Polar mapping data were used to define the borders of V1, used as a seed ROI for the RSFC analyses. Control seed ROIs included anatomically defined Brodmann areas (from the anatomic atlas in BrainVoyager) with the exception of visual association areas BA 18, 19, and 37. BAs 18 and 19 were tested separately (Extended Data Fig. 1-3).

### RSFC variability analyses

**Figure 1.**
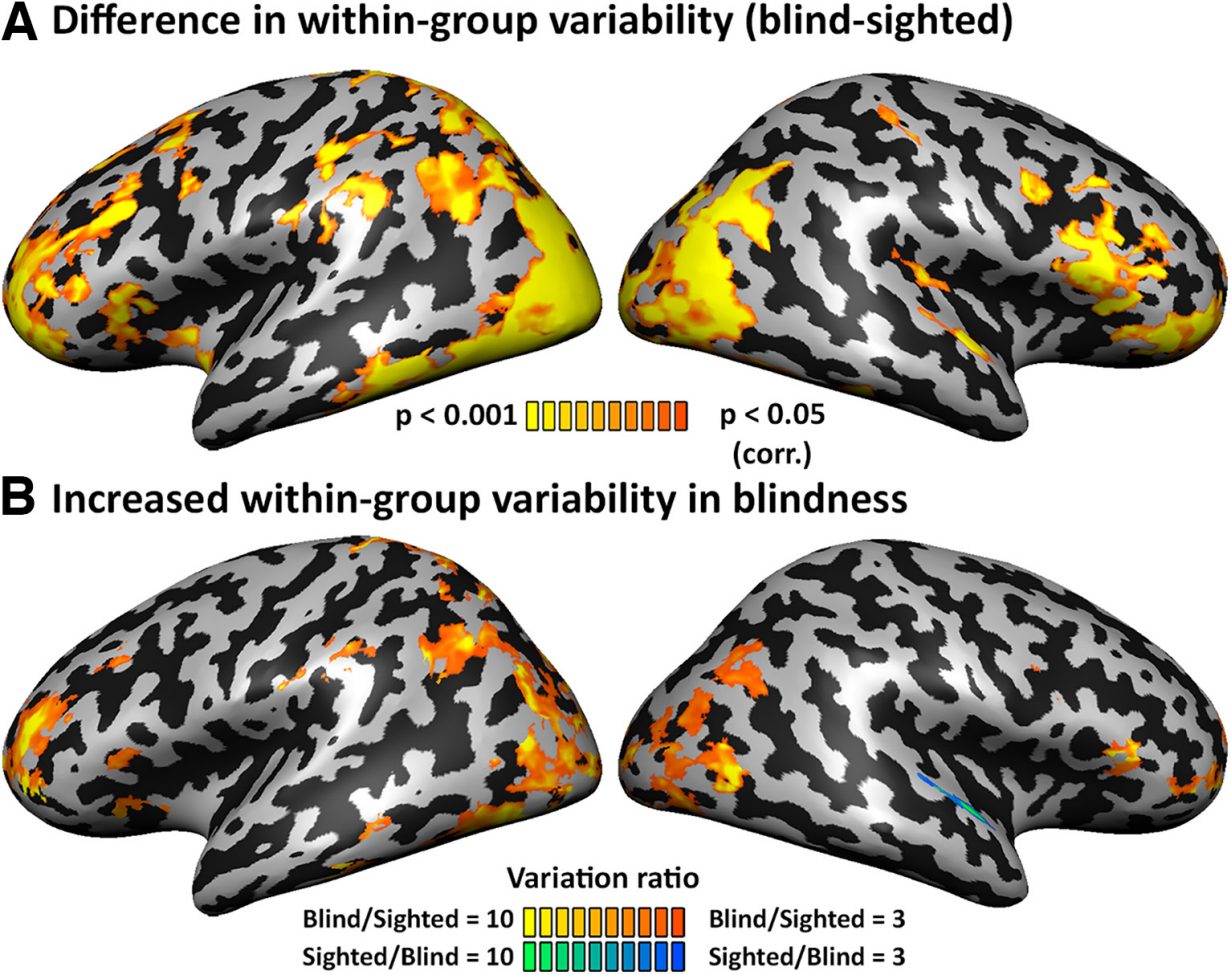
Variability in brain connectivity is increased in blindness. ***A***, The difference in within-group variability between the groups is significant in various parts of the brain, including in the frontal lobe. ***B***, Directional comparison of the within-group variability difference (ratio of blind intragroup variability divided by sighted intragroup variability >3) shows that the blind have increased variability in most of the regions differing in their variation between the groups. This suggests a stabilizing effect of visual experience on visual cortex developmental functional connectivity. Extended Data Figure 1-1 shows a replication of the results with global signal regression. Extended Data Figure 1-2 shows a replication of the results when controlling for the increased mean RSFC in blindness and for V1 cortical thickness. When controlling for increased mean V1-RSFC in blindness, the variability difference between the groups is even more robust and covers larger portions of cortex (***A***). Importantly, almost all the variability difference shows increased variability in blindness (***B***), supporting the conclusion that individual differences increase in blindness. Extended Data Figure 1-3 shows comparable analyses for association visual cortex Brodmann areas 18 and 19.

**Figure 2.**
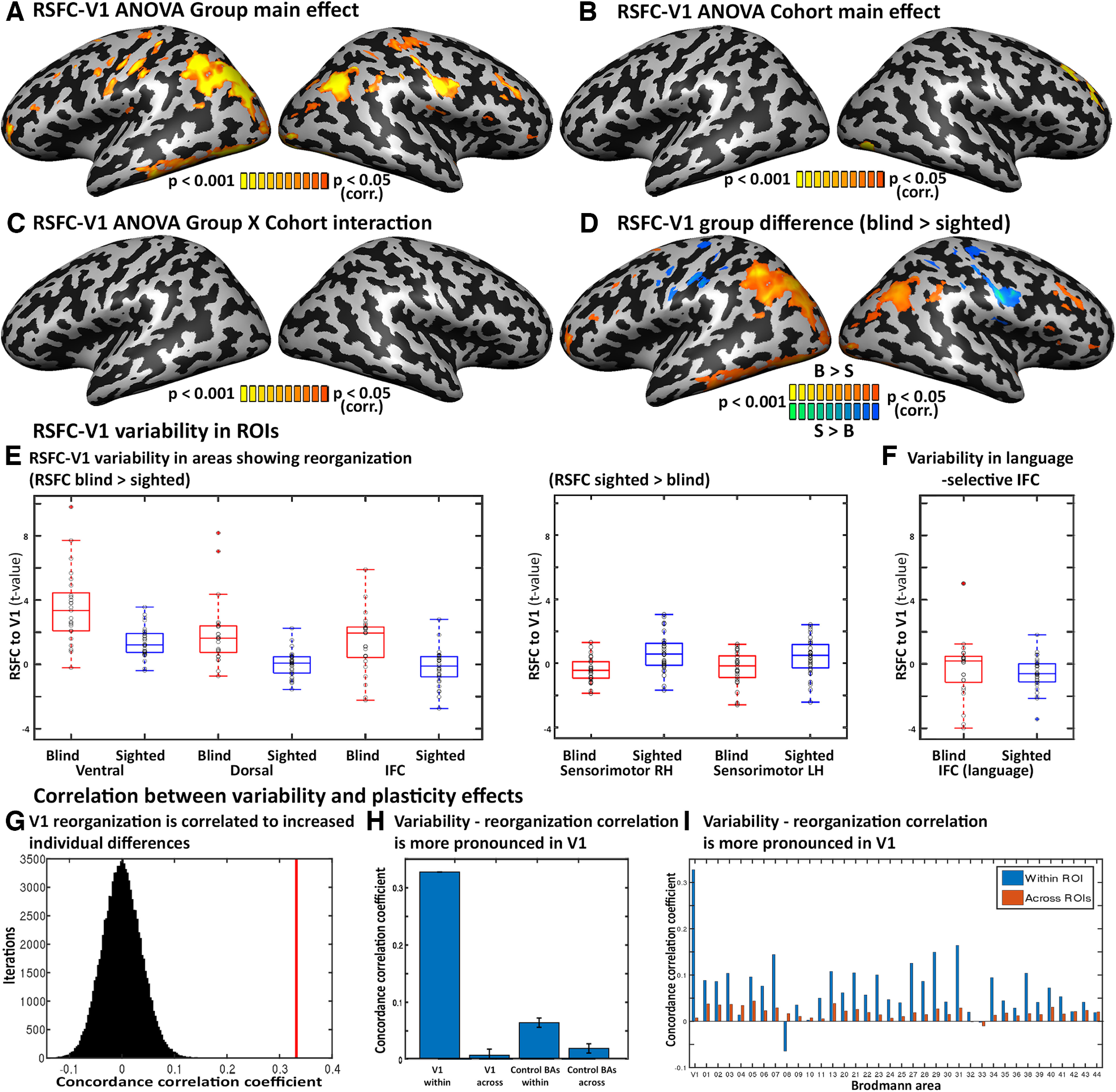
Brain reorganization in blindness is associated with increased interindividual variability. ***A-C***, Main effects and interactions for the Group X Cohort ANOVA for V1-RSFC. ***A***, The effect of sight across the cohorts is depicted. As reported before, the blind and sighted differed in their RSFC from the primary visual cortex to visual, parietal, and frontal regions. ***B***, The main effect of cohort across the groups, showing little difference focused in the right superior frontal cortex. ***C***, The Group X Cohort interaction shows no significant effect. ***D***, Increased V1-seeded RSFC in blindness is found in the visual streams, as well as in the bilateral IFC. ***E***, ***F***, The blind show increased variability in their V1-seeded RSFC to left ventral stream, dorsal stream, and IFC frontal areas. Box plots are presented for the blind and sighted in red and blue, respectively. The central mark indicates the median, and the bottom and top edges of the box indicate the 25th and 75th percentiles, respectively. Error bars represent the standard deviation. Individual participant data are presented in circles. ***E***, Within the areas showing increased RSFC in the blind (Fig. 2*D*). The sensorimotor cortex, showing decreased V1-RSFC in blindness, showed similar but slightly lower variability in the blind. ***F***, The blind show increased variability in their V1-seeded RSFC to a language-selective IFC ROI, defined by preference toward words compared with pseudowords. ***G***, Overall across the brain, areas showing changes in RSFC in blindness also show increased variability across blind participants. The concordance correlation coefficient was calculated between the RSFC group difference and RSFC change in variability for the V1 seed (red line) and compared with a spatial permutation test (distribution in black). ***H***, The link between reorganization and increased variability in blindness is more pronounced in V1. Correlation between the two maps for the V1 seed was significantly greater than in correlating across seeds and significantly greater for V1 compared with other nonvisual Brodmann areas. Error bars represent the standard deviation. ***I***, The link between reorganization and increased variability in blindness is presented for V1 (bar, far left) and all control nonvisual Brodmann areas in blue. For each area, the across-seed correlation is shown in red. The within-seed correlation for all control areas was lower than for V1; however, the comparison between within- and across-seed correlation was significant, suggesting that more broadly, reorganization manifests in greater variability.

Individual time courses from the V1 seed ROI were sampled from each of the participants, *z* transformed and used as individual predictors in a *z*-normalized GLM analysis, with individual motion estimates (six degrees of freedom and their first derivatives) as nuisance predictors. Individual RSFC maps were spatially smoothed with a 6 mm full-width-at-half-maximum Gaussian kernel for group analyses. Data were analyzed with a 2 × 2 random effects ANOVA (Group {blind, sighted} × Cohort {A,B}) at the voxel level. In addition to the main effect of Group (see Fig. 2*A*; Fig. 2*B*,*C*, showing limited cohort effect and group X cohort interaction), we calculated the Brown–Forsythe test for equal variance for this main effect, testing whether the two groups differed in their interindividual variability of the RSFC values (Fig. 1*A*). The Brown–Forsythe test (Brown and Forsythe, 1974) is a homogeneity of variance test similar to Levene's test, conventionally used to test for variability differences, but uses the median instead of the mean, safeguarding against false positives in cases of skewed data distribution (Olejnik and Algina, 1987). The same analyses were performed for all nonvisual control seed ROIs (Brodmann areas) for the comparison of variability and reorganization correlation (details below). The minimum significance level of all results presented in this study was set to *p* < 0.05, corrected for multiple comparisons within the gray matter volume using the spatial extent method (a set-level statistical inference correction; Friston et al., 1994; Forman et al., 1995). Correction was based on the Monte Carlo simulation approach, extended to 3D datasets using the threshold size plug-in for BrainVoyager QX. We additionally computed the variability of RSFC within each group separately, using normalized data of each group to overcome possible effects of the different cohorts on the mean and SD of the RSFC. To inspect the direction of the variability group effect, we computed the ratio of variability between the groups (Variability_Blind_/Variability_Sighted_; Fig. 1*B*) for each voxel showing a significant Brown–Forsythe test effect (*p* < 0.05, corrected). The same calculation of the variability ratio was also conducted within several ROIs tested for their increased variability (details below).

To inspect the direction of reorganization in V1 RSFC, in addition to the ANOVA model of the main effect of group on V1-RSFC (Fig. 2*A*), we computed a *post hoc t*-test comparing RSFC between the groups (blind vs sighted; Fig. 2*D*).

To quantitatively assess the link between reorganization in the blind and variability effects, we compared the spatial pattern of variability (Fig. 1*A*) and reorganization in the blind (Fig. 2*A*), by computing the concordance correlation coefficients (CCC; Lin, 1989) between these maps, within the gray matter. CCCs were computed using custom software written in MATLAB (MathWorks). Concordance correlation Although values range from 1 (perfect spatial similarity) to −1 (perfect spatial dissimilarity). While CCC, similarly to Pearson's linear correlation coefficient, tests for shared fluctuations in variance of two datasets, it also penalizes for differences in means between the two sets, thus serving as a more sensitive measure for map differences in both spatial patterns and overall values. The significance level for the CCCs was obtained using a permutation test (100,000 iterations) randomly shuffling voxels from one map and convolving the resulting map with a Gaussian kernel based on data smoothness estimation to account for spatial autocorrelation. As an additional control, we compared the CCC values across regions of interest for pairs of maps (a variability map and a group-difference map) stemming from a coupled comparison for the same-seed ROI as compared with correlation values stemming from comparisons of variability and group-difference maps across seed ROIs. For example, computing the CCC between the Brown–Forsythe test map for the V1 seed and the map of blind-sighted group effect for the same seed as compared with the CCC between the Brown–Forsythe test for the V1 seed and the map of blind-sighted group effect for each of the other nonvisual Brodmann area seed ROIs. Statistically testing the difference between same-seed comparison and an across-seed comparison, following Fischer transformation of the R values, allowed us to examine the specificity of the found comparison to V1.

### Mean normalization and correlation with cortical thickness controls

To verify our findings of increased variability do not stem from the increased RSFC from V1 in blindness, we replicated the variability analysis when subtracting from each participant's V1-RSFC map the mean of its group and cohort (i.e., subtracting the mean of the cohort A blind group from each of the participants from this subgroup). This analysis showed that even when mean connectivity is controlled, variability is significantly higher in the blind (Extended Data Fig. 1-2*A*,*B*). As cortical thickness is increased in blindness (Bridge et al., 2009; Jiang et al., 2009) and correlates to cross-modal activation (Voss and Zatorre, 2011; Anurova et al., 2015; Aguirre et al., 2016), we also verified that our results cannot be attributed to cortical thickness variability. FreeSurfer version 7.2.0 (Fischl, 2012) was used to automatically parcellate cortical and segment subcortical brain regions from T1-weighted anatomic images. Cortical parcellations were identified and labeled within a surface-based processing stream, sampling cortical thickness from the V1-exVivo section (Desikan et al., 2006; Hinds et al., 2008). We then calculated the correlation between the V1-seeded RSFC of each voxel for all participants with their V1 thickness values. No correlation to cortical thickness was found even at an extremely lenient threshold (Extended Data Fig. 1-2*C*), showing that the increased variability in connectivity is not explained by cortical thickness.

### Variability ROI analysis

Given the proposal that increased connectivity with the frontal cortex (Liu et al., 2007; Hawellek et al., 2013; Burton et al., 2014; Deen et al., 2015; Striem-Amit et al., 2015; Abboud and Cohen, 2019) drives reorganization in the visual cortex of the blind (Deen et al., 2015; Bedny, 2017; Abboud and Cohen, 2019; Rimmele et al., 2019), we inspected the variability of the V1-seeded RSFC to the frontal lobe, using two sampling ROIs, (1) clusters in the inferior frontal lobe showing increased functional connectivity from V1 in the blind in the present study (Fig. 2*D*) and (2) a left-lateralized language-selective region in the inferior frontal cortex (Talairach coordinates −29, 15, 18), defined from a contrast of heard object names greater than heard pseudowords in the joint group of blind and sighted subjects from cohort B. The full experimental protocol for this contrast is detailed in Striem-Amit et al. (2018b); briefly, auditory pseudowords and words from different concept categories were presented in a block-design fMRI experiment. Additional regions of interest showing increased or decreased functional connectivity from V1 in the blind in the present study (Fig. 2*D*) were also tested. These included the ventral and dorsal visual cortex clusters (showing V1-RSFC blind > sighted) and sensorimotor cortex (showing V1-RSFC sighted > blind). For all ROIs, we sampled V1 RSFC GLM t-values of parameter estimates (betas) from each of the participants, and the variability (S^2^) of each group was calculated as well as the ratio between them to assess whether the groups differed in their intragroup variabilities.

### Clustering analysis

**Figure 3.**
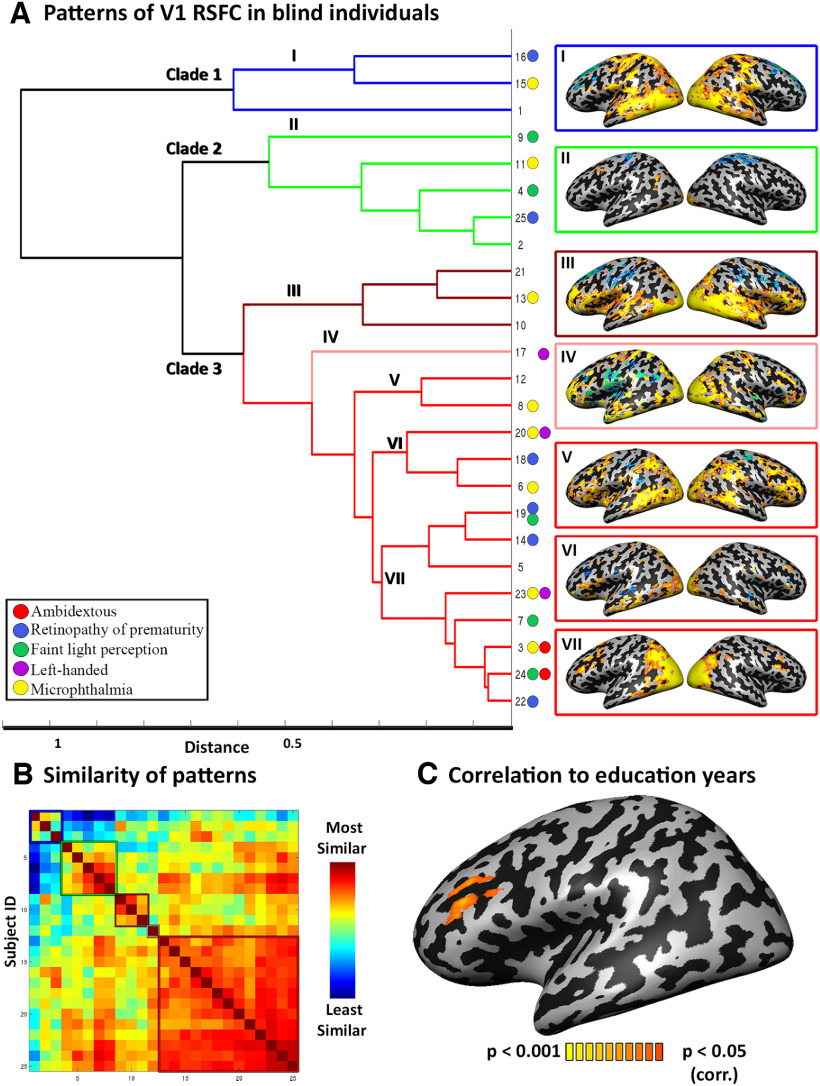
Patterns of brain reorganization in blindness. ***A***, V1-RSFC of each individual blind participant to each Brodmann area was used to compute hierarchical clustering of RSFC patterns across the blind. Three main clades emerge, with differential connectivity to sensorimotor and frontal cortices. Subclades are marked with Roman numerals, and an average V1-RSFC map for the individuals in each subclade is shown. Color circles by participant numbers indicate frequent blindness etiologies (Retinopathy of prematurity - ROP, blue; microphthalmia, yellow) and unique behavioral traits (ambidextrous individuals, red; left-handedness, purple; and faint light perception as opposed to no light perception, green). Hierarchical clustering in the blind does not support linking blindness etiology or crude light perception to the similarity in V1 RSFC profile. With the exception of the two ambidextrous individuals being clustered together, no other qualitative pattern is evident linking blindness etiology or light perception to the similarity in V1 RSFC profiles. Participants 13 and 20, found on different subclades, are siblings who are blind because of genetic microphthalmia. Extended Data Figure 3-1 shows comparable hierarchical clustering in the sighted group, showing lower distances than in the blind. ***B***, The V1-RSFC correlation (similarity) structure between individuals based on which hierarchical clustering analysis was conducted. ***C***, V1-RSFC to the left inferior frontal cortex in the blind (and not in the sighted) is correlated to the duration of formal education, showing one environmental factor affecting individual differences in brain reorganization in blindness.

To qualitatively explore individual differences in the RSFC from the visual cortex of the blind, we performed a hierarchical clustering analysis across subjects' V1-seeded RSFC maps, using RSFC values for each individual from each of the Brodmann areas in the BrainVoyager atlas (see above). Distance was calculated as the correlation between individual RSFC vectors, implemented in MATLAB (MathWorks). A dendrogram of the distances across all participants was computed based on complete distance between clusters (Fig. 3*A*; Fig. 3*B* shows the underlying correlation dissimilarity matrix). As a preliminary quantitative exploration of the clustering analysis, the average RSFC pattern (average V1-RSFC *t* map across the subjects) for individuals within each subclade was computed. The hierarchical clustering was also similarly conducted on individual maps derived from sighted participants. The distance values of lower nondiagonal elements of the dissimilarity matrix were statistically compared between the groups.

### Correlation with education

As a preliminarily analysis to inspect the effect of specific environmental factors on V1 RSFC variability, we calculated the correlation between the V1-seeded RSFC of each voxel for all participants with the number of years of formal schooling they received for each group separately at the whole-brain level using a gray matter mask at *p* < 0.05 corrected (Fig. 3*C*, for the blind; the sighted showed no significant correlation). In the IFS cluster showing such correlation in the blind, correlation in the sighted group was also sampled.

### Data availability

Study data are available on request from the corresponding author.

## Results

### V1 variability differs between congenitally blind and sighted individuals

We tested whether visual deprivation leads to altered interindividual variability in the connectivity patterns of the primary visual cortex in a large group of congenitally fully blind adults (*n* = 25; Table 1) and sighted adults (*n* = 31) from two experimental cohorts scanned previously (Striem-Amit et al., 2015, 2018b; each cohort contained a blind and matched sighted group). We computed RSFC from an anatomically defined seed in retinotopic primary V1, based on a visual localizer in an independent group of sighted individuals (Striem-Amit et al., 2015). To assess whether RSFC variability effects are indeed because of the absence of shared experience, the same procedures were computed for control seed regions in all nonvisual Brodmann areas.

We first tested whether there are differences in interindividual variability of the V1-seeded RSFC resulting from blindness. For this aim, RSFC maps were analyzed using ANOVA (cohort times group, to remove any cohort effects, in addition to relevant preprocessing steps; see above, Materials and Methods). As the cohort differences were negligible and highly localized (Fig. 2*B*,*C*), RSFC maps across cohorts within each group (blind, sighted) were analyzed for their voxel-wise variability across individuals. We calculated a whole-brain voxel-level homogeneity of variance test (Brown–Forsythe test; Brown and Forsythe, 1974; see above, Materials and Methods) for the group main effect, testing whether the two groups differed in their interindividual variability of the RSFC values. This analysis revealed multiple areas that exhibit a significant intersubject difference in V1-seeded RSFC variability between the blind and sighted groups (Fig. 1*A*; group variability difference). These included areas of the ventral and dorsal visual pathways, posterior inferior parietal cortex, and the inferior frontal cortex. Therefore, visual experience affects brain consistency. This analysis reveals only a nondirectional difference in variability; to directly test the sign of the group difference, we calculated the ratio of variability between the groups (blind/sighted) across the brain (Fig. 1*B*, ratio shown within areas that differ in variability between the groups). It is apparent that the blind show higher variability than the sighted in multiple areas, including parietal and frontal regions, with lower variability in only one cluster in the right auditory cortex. Thus, visual experience can have an overall stabilizing effect on RSFC, and visual deprivation results in overall more variable RSFC from the visual cortex. This suggests a role of shared experience in promoting consistency of neural organization.

10.1523/JNEUROSCI.1700-21.2022.f1-1 Figure 1-1 Reproducing main analyses with global signal regression as a preprocessing step. Main analyses were reproduced when including global signal regression as a preprocessing step. ***A***, Replicating Figure 1*A*, the difference in within-group variability between the groups is significant in various parts of the brain, including in the frontal lobe. ***B***, Replicating Figure 1*B*, directional comparison of the within-group variability difference (ratio of blind intragroup variability divided by sighted intragroup variability >3) shows that the blind have increased variability in most of the regions differing in their variation between the groups. This suggests a stabilizing effect of visual experience on visual cortex developmental functional connectivity. ***C***, Replicating Figure 2*D*, increased V1-seeded RSFC in blindness is found in the visual streams as well as in the bilateral IFC. Global signal regression is known to introduce anticorrelation, distortion of group differences, and exacerbation of distance-dependent motion artifacts (Murphy et al., 2009; Anderson et al., 2011; Power et al., 2012; Saad et al., 2012; Satterthwaite et al., 2012; Gotts et al., 2013; Hahamy et al., 2014; Ciric et al., 2017), thus this somewhat quantitatively differs from Figure 2*D* for group differences distant from the seed ROI. ***D***, ***E***, Replicating Figure 2, *E* and *F*. The blind show increased variability in their V1-seeded RSFC to left ventral and dorsal streams and IFC frontal areas as well as decreased connectivity to V1 in the sensorimotor cortex. ***E***, Within the areas showing increased RSFC in the blind (Extended Data Fig. 1-1*C*). ***F***, Sampled in a language-selective IFC ROI, defined by preference toward words compared with pseudowords. Box plots are presented for the blind and sighted in red and blue, respectively. The central mark indicates the median, and the bottom and top edges of the box indicate the 25th and 75th percentiles, respectively. Individual participant data are presented in circles. ***F***, Replicating Figure 2*G*. Overall, across the brain, areas showing changes in RSFC in blindness also show increased variability across blind participants. The concordance correlation coefficient was calculated between the RSFC group difference and RSFC change in variability for the V1 seed (red line) and compared with a spatial permutation test (distribution in black). ***G***, Replicating Figure 2*H*, the link between reorganization and increased variability in blindness is more pronounced in V1. Correlation between the two maps for the same seed was significantly greater than in correlating across seeds and significantly greater for V1 as compared with other nonvisual Brodmann areas. ***H***, Replicating Figure 3*C*, V1-RSFC to the left-lateralized inferior frontal cortex (sampled from the cluster in Fig. 3*C*) in the blind (left, red; not in the sighted, right, blue) is correlated to the duration of formal education in the blind only (*r*_(25)_ = 0.58, *p* = 0.0025; sighted, *r*_(31)_ = −0.14, *p* = 0.45), showing one environmental factor affecting individual differences in brain reorganization in blindness. The correlation was not sufficiently robust to appear in a cluster-corrected whole-brain analysis. ***I***, Replicating Extended Data Figure 1-2*A*, the difference in variability between the groups is significant in various parts of the brain, including in the frontal lobe, even when controlling for the higher mean RSFC values in the blind. ***J***, Replicating Extended Data Figure 1-2*B*, the blind show increased variability (ratio of blind intragroup variability divided by sighted intragroup variability >10) in most of the regions differing in their variation between the groups, when controlling for the higher mean RSFC values in the blind. ***K***, Replicating Extended Data Figure 1-2*C*, V1 cortical thickness, showing a difference between the groups, is not correlated to the functional connectivity from V1 in the blind (or across both groups, also showing no significant effect), even at a lenient threshold of *p* < 0.01 uncorrected, and can therefore not account for the change in connectivity variability. Download Figure 1-1, TIF file.

10.1523/JNEUROSCI.1700-21.2022.f1-2 Figure 1-2 Variability increase in blindness is found regardless of changes to mean RSFC. ***A***, The difference in variability between the groups (replication of the analysis shown in Fig. 1*A* when demeaning the V1-RSFC maps) is significant in various parts of the brain, including in the frontal lobe, even when controlling for the higher mean RSFC values in the blind. This difference in variability is even more robust, compared with Figure 1*A*, and covers larger portions of cortex. ***B***, The blind show increased variability (ratio of blind intragroup variability divided by sighted intragroup variability >10; replication of the analysis shown in Figure 1*B* when demeaning the V1-RSFC maps) in the vast majority of the regions differing in their variation between the groups when controlling for the higher mean RSFC values in the blind. This supports the conclusion that individual differences increase in blindness. ***C***, V1 cortical thickness, showing a difference between the groups, is not correlated to the functional connectivity from V1 in the blind (or across both groups, also showing no significant effect), even at a lenient threshold of *p* < 0.01 uncorrected, and can therefore not account for the change in connectivity variability. Download Figure 1-2, TIF file.

10.1523/JNEUROSCI.1700-21.2022.f1-3 Figure 1-3 Variability changes in functional connectivity to association visual cortex. ***A–C***, Analyses of variability are presented for visual areas V1 (***A***, replicating analyses in the article), Brodmann area 18 (***B***), and Brodmann area 19 (***C***). Left column, Analyses of the difference in within-group variability between the blind and sighted (replicating Fig. 1*A* for V1) show changes to variability because of blindness are not limited to V1. Second column, A main effect of sight across the cohorts is depicted. (replicating Fig. 2*A* for V1). Third column, Directional comparison of the within-group variability difference (ratio of blind intragroup variability divided by sighted intragroup variability >3) shows that the blind have increased variability in most of the regions differing in their variation between the groups (replicating Fig. 1*B* for V1). Far right, The concordance correlation coefficient was calculated between the RSFC group difference and RSFC change in variability for the seeds (red line) and compared with a spatial permutation test (distribution in black). Only V1 shows correlation between the two effects (replicating Fig. 2*G*), whereas BA18 and BA19 do not (BA18 CCC = 0.01, *p* = 0.81, BA19 CCC = −0.003, *p* = 0.94). Download Figure 1-3, TIF file.

### V1 variability increases especially for areas that reorganize in blindness

Inspecting interindividual variability also allowed us to test whether neural reorganization is consistent across blind individuals. Are areas whose connectivity and function have reorganized because of blindness also highly variable among blind individuals compared with the typical interindividual differences for these areas? We tested this by inspecting the intragroup variability difference in the areas showing a main effect of group in the V1-RSFC values; areas showing change in V1-seeded RSFC between the blind and sighted (a two-way ANOVA main group effect; Fig. 2*A*; Extended Data Fig. 1-1 shows replication of the results with global signal regression).

In accordance with previous work (Liu et al., 2007; Yu et al., 2008; Wang et al., 2014; Burton et al., 2014; Qin et al., 2015; Striem-Amit et al., 2015), group differences in functional connectivity were robust (Fig. 2*A*). Blind individuals showed increased functional connectivity to some regions in the visual cortex and several areas in the frontal lobe, including the inferior frontal sulcus (Fig. 2*D*). We sampled the areas showing a V1-RSFC group difference to inspect whether they would also show increased variability in the blind group. Indeed, variability of the RSFC to large regions in the ventral and dorsal streams was five times greater in blindness (Fig. 2*E*; ventral stream, S^2^_sighted_ = 0.86, S^2^_Blind_ = 5.13; dorsal stream, S^2^_sighted_ = 0.64, S^2^_Blind_ = 4.15). Curiously, variability of RSFC to the sensorimotor cortex, which showed reduced functional connectivity to the visual cortex in blindness was slightly decreased in the blind (left hemisphere, S^2^ blind = 1.05, S^2^ sighted =1.30, variance ratio 0.81; right hemisphere, S^2^ blind = 0.63, S^2^ sighted = 1.34; variance ratio 0.47), although the difference did not reach significance (Brown–Forsythe test, left hemisphere, *F* = 0.75; right hemisphere, *F* = 0.96; Fig. 2*E*).

Given the proposal that increased connectivity with the frontal cortex (Liu et al., 2007; Hawellek et al., 2013; Burton et al., 2014; Deen et al., 2015; Striem-Amit et al., 2015; Abboud and Cohen, 2019) drives reorganization in the visual cortex of the blind (Deen et al., 2015; Bedny, 2017; Abboud and Cohen, 2019; Rimmele et al., 2019), we tested RSFC variability in these foci within the group of blind participants. Inferior frontal areas that show increased RSFC in the blind show more than double the variability within the blind group as within the sighted group (Fig. 2*E*; S^2^_sighted_ = 1.21, S^2^_Blind_ = 3.45). To specifically test frontal regions proposed to affect visual cortex reorganization, we directly examined the variability of connectivity in left-lateralized frontal language regions. A spoken-language-selective region was defined in the left inferior frontal sulcus (from a contrast of responses to heard object names more than to heard pseudowords in a joint group of blind and sighted subjects from Striem-Amit et al., 2018b; see above, Materials and Methods). In this region as well, the intrablind RSFC-with-V1 variability was more than quadruple the intrasighted variability (S^2^_sighted_ = 1.00, S^2^_blind_ = 4.39; Fig. 2*F*). Therefore, it appears that reorganization in the connectivity between the visual and frontal cortex in the blind is highly variable among the blind individuals.

Is this a general pattern, that neural reorganization manifests more variably in blindness? We correlated the spatial pattern of the group difference in mean RSFC from the visual cortex seed (Fig. 2*D*) with the variability difference between the groups (Fig. 1*B*, computed within a gray matter mask). The concordance correlation coefficient between the two maps (Lin, 1989) was highly significant (CCC = 0.332, *p* < 0.00,001; using a permutation test shuffling the order of the voxels, 100,000 iterations; Fig. 2*G*). Therefore, it appears that when the brain reorganizes, it introduces a further source of variance, resulting in more diverse connectivity values. Importantly, the link between reorganization and variability is not an artifact because of the higher mean difference between the groups. Using group-normalized V1-RSFC values shows that the variability is increased in the blind even when controlling for the higher group mean value (Extended Data Fig. 1-2*A*,*B*) and when regressing out the global signal (Extended Data Fig. 1-1).

Next, we tested the specificity of the link between reorganization and increased variability. If this pattern is driven by visual deprivation, we expected it to be especially prominent for the primary visual cortex seed, compared with seeds in nonvisual areas. As a control, we performed the same analysis we performed on V1 in a whole-brain level via parcellation to Brodmann areas and used each of the nonvisual Brodmann areas (with the exception of areas 17, 18, 19, and 37) as a seed for RSFC variability analyses. Nonvisual regions did not show the same phenomena as V1. Specifically, there was a significantly less pronounced change to the variability of functional connectivity between the groups from nonvisual seed ROIs as compared with V1 (comparing number of significant voxels showing a significant variability change; *t*_(34)_ = 21.55, *p* < 0.0001). It is important to note that given the increased variability of connectivity from the early visual cortex to most other cortical areas, we expected a nonzero change in variability in nonvisual areas as well because their connectivity to at least the primary visual cortex is expected to increase. Moreover, nonvisual Brodmann areas did not show as significant a link between increased variability and reorganization. The correlation between the Brown–Forsythe map and the ANOVA main group effect was significantly lower than the corresponding correlation for V1 (*t*_(34)_ = 60.97, *p* < 0.0001). Further, we performed the correlation analysis between the group difference for V1 and the variability difference across the different seeds as a permutation test. The cross-seed correlation, the correlation between the group difference for V1 and the variability difference of any other Brodmann area computed in a gray matter mask, was close to zero (CCC = 0.0017; Fig. 2*H*), showing that the link between variability and reorganization is spatially specific. However, the difference between matched and permuted, cross-seed correlations was also significant for the nonvisual Brodmann areas (*t*_(68)_ = 5.34, *p* < 0.0001; Fig. 2*I*). This shows that although the link between the increase in variability and change in RSFC in the blind is much more pronounced in connectivity with the visual cortex, even more broadly, reorganization is correlated to greater variability. Overall, this suggests that visual cortex plasticity is characterized by increased variability and not by a ubiquitous change for all individuals.

### Spatial patterns variability across blind individuals

What forms does this increased variability take? We further asked whether the plastic reorganization of visual cortex functional connectivity (Liu et al., 2007; Yu et al., 2008; Wang et al., 2014; Deen et al., 2015; Striem-Amit et al., 2015; Abboud and Cohen, 2019) manifests in a stereotypical, similar change across blind individuals, or if it is spatially idiosyncratic. To inspect whether variability also manifests in different spatial patterns of connectivity in the blind, we used hierarchical clustering to group the blind individuals into clades based on their RSFC patterns and examined the RSFC pattern characterizing each subclade. This approach revealed informative diversity in the profiles of RSFC of the visual cortex among the blind individuals (Fig. 3*A*; Fig. 3*B* shows the correlation matrix underlying this clustering). Most of the blind individuals clustered together in a clade showing (on average) focused positive RSFC with foci in the inferior frontal cortex (IFC; clade 3, 17 individuals), along with differential patterns of RSFC with the superior frontal lobe: positive and negative values across individuals in different subclades (e.g., subclades III and V). Curiously, in most of these subclades, RSFC to the IFC was bilateral (subclades V and VII), whereas in a subclade of three individuals the pattern seemed lateralized to the left IFC (Fig. 3*A*, subclade VI). Given that functional connectivity to the left frontal cortex is the most drastic form of connectivity reorganization associated with blindness (Liu et al., 2007; Yu et al., 2008; Wang et al., 2014; Burton et al., 2014; Qin et al., 2015; Striem-Amit et al., 2015), which has been described driving it toward functionally processing language (Bedny, 2017), the rarity of its lateralization in blind individuals is curious.

10.1523/JNEUROSCI.1700-21.2022.f3-1 Figure 3-1 V1 RSFC variability is affected by blindness. V1-RSFC of each individual participant to each Brodmann area was used to compute hierarchical clustering of RSFC patterns within each group. Left, Replication of Figure 3*A* showing the clustering of the blind into three main clades and subclades. Right, The comparable clustering dendrogram in the sighted group. Although division to a similar number of clades and subclades can be found, the overall distances were lower, as evident by the *y*-axis, and the distances were significantly smaller than in the blind group (*t*_(52)_ = 3.17, *p* = 0.007). Download Figure 3-1, TIF file.

Two additional smaller clades seemed to cluster separately based on RSFC with the sensorimotor and auditory cortices, with a small clade (clade 2, five individuals) showing negative RSFC (anticorrelation) with the sensorimotor cortex, and three individuals (clade 1) showing a pattern of positive RSFC with the sensorimotor cortex as well as with the auditory cortex. Although the sighted data also yielded a similar number of clades, its overall distances were lower (*t*_(52)_ = 3.17, *p* = 0.007; Extended Data Fig. 3-1). Interestingly, the clustering in the blind did not show any qualitative distinction based on blindness etiology (Fig. 3*A*), including a sparse distribution among clades for individuals whose blindness stemmed from genetic causes such as microphthalmia. Together, this analysis revealed a diverse pattern of organization relative to the visual cortex across blind individuals.

Can we identify specific environmental factors contributing to this spatial diversity across blind individuals? As a supplementary analysis, we computed the correlation between V1-seeded RSFC and one socioeconomically dependent factor, that is, each individual's years of formal education. We anticipated that visual cortex connectivity may be influenced by this factor because the visual cortex of the blind has been implicated in language (Burton et al., 2003; Amedi et al., 2004; Bedny et al., 2011; Abboud and Cohen, 2019), memory (Amedi et al., 2003; Raz et al., 2005; Abboud and Cohen, 2019), numerical thinking (Kanjlia et al., 2016), and executive function (Deen et al., 2015; Abboud and Cohen, 2019), all functions that are trained in formal education. Indeed, this was the case. V1-seeded RSFC with a region in the left inferior frontal cortex (dorsolateral prefrontal cortex) was the only one correlated in a whole-brain analysis with education years in the blind group (Fig. 3*C*; *p* < 0.05 corrected; peak Talairach coordinates, −36, 26, 24, *r* = 0.71, *p* = 0.00006). The sighted showed no correlation between years of education and RSFC from V1 to any brain region (no significant clusters; whole-brain analysis, *p* < 0.05 corrected), including in the IFC clusters showing such correlation in the blind (sampled as an ROI in the sighted; *r* = −0.078, *p* = 0.68). Curiously, the IFC area, which showed correlation to education duration, is found in close proximity and partly overlaps with areas showing increased variability between the blind and sighted, as well as increased RSFC in the blind group as compared with the sighted. Therefore, this exemplifies an interaction of blindness with environmental life circumstances that affects the diversity of visual cortex reorganization.

## Discussion

Interindividual differences in brain organization stem from both hereditary and environmental factors. Here, we examined the role of one extreme environmental factor, lack of visual experience, on the variability of the functional connectivity with the primary visual cortex. We showed that interindividual differences in connectivity are higher in blind individuals (Fig. 1*B*), suggesting that shared sensory experience enforces consistency across individuals. Brain network variability is expanded in its absence. Furthermore, we found that areas showing reorganization because of blindness, manifesting as increased RSFC with V1, also showed increased variability among blind individuals (Fig. 2). This intragroup variability suggests that plasticity is not uniform among the blind, generating more variable outcomes than is typical in sighted individuals. We qualitatively demonstrated different spatial patterns that variable reorganization takes by characterizing reorganization in distinct subgroups of blind individuals (Fig. 3*A*). Although functional connectivity to the left frontal lobe has been described as a key characteristic of plasticity in blindness, we found that only some blind individuals show this pattern. Functional connectivity between the visual cortex and inferior frontal cortex (potentially related to language and working memory; Rottschy et al., 2012) was correlated with the duration of formal education, supporting a role for both sensory and social-educational postnatal factors in acquiring brain variability in blindness (Fig. 3*C*). These findings inform the developmental origins of individual variability, the properties of brain plasticity in blindness and beyond, and the importance of considering variability for the rehabilitation of visual loss. In the following sections, we address all these topics in more depth.

Brain connectivity allows identifying individual fingerprints (Finn et al., 2015; Gratton et al., 2018) correlated with behavioral capacities (Koyama et al., 2011; Baldassarre et al., 2012; Vaidya and Gordon, 2013; Wang et al., 2018; Fong et al., 2019). These may form quantitative phenotypes in molecular and genetic studies of neurologic and psychiatric diseases and guide medical interventions (Biswal et al., 2010; Fox and Greicius, 2010; Rosenberg et al., 2016; Drysdale et al., 2017; Xin et al., 2019). Importantly, individual connectivity differences are stable over time (Chou et al., 2012; Jovicich et al., 2016; Badhwar et al., 2020), suggesting they reflect true anatomic and functional differences rather than merely temporary scan-time cognitive states. However, the contributing factors underlying this variability are not clear. A role for inherited genetic components of neural variability is evident (Thompson et al., 2001; Polk et al., 2007; Koten et al., 2009; Park et al., 2012a, b; Gao et al., 2014; Jansen et al., 2015; Ge et al., 2017; Xin et al., 2019; Anderson et al., 2021), including specific genetic components underlying variability in multisensory connectivity in blind children (Ortiz-Terán et al., 2017). However, a better understanding of the environmental components is needed. Developmental studies highlight the adverse effects of social-environmental deprivation on children and adolescents (Gunnar and Reid, 2019; Herzberg and Gunnar, 2020). This emphasizes the importance of understanding plasticity through the lens of individual differences.

Here, we studied the role of a more extreme form of environmental change—complete deprivation of an entire sensory channel. We showed that experience has immense effects on individual differences and can modify the variability in the neural connectivity profile of extensive cortical tissue. In the past, functional connectivity variability was found to be highest in association cortices that developed phylogenetically recently (Kaas, 2006; Smaers et al., 2011; Krubitzer and Prescott, 2018), whereas sensory cortices exhibited low variability (Fischl et al., 2008; Mueller et al., 2013; Xu et al., 2018; Anderson et al., 2021). However, studying typically developed individuals does not allow us to resolve whether increased individual variability in these regions results from longer exposure to environmental factors in the individual's lifetime or from less tight genetic control for later developed phylogenetic regions allowing more diversity, as the two factors are typically confounded. Association networks develop through adolescence, whereas early sensorimotor systems mature earlier (Guillery, 2005; Shaw et al., 2008; Raznahan et al., 2011; Amlien et al., 2016; Xu et al., 2018). Our study shows how experience can affect even an evolutionarily conserved typically highly consistent cortical area, whose connectivity typically stabilizes in early childhood (Xu et al., 2018). Furthermore, although the direction of the correlation cannot be directly inferred, we showed how a social-environmental factor, years of education, which extends into adulthood, could correlate to the variability of RSFC in V1. Given the considerable barriers blind individuals face to complete higher education (Tielsch et al., 1991; Klein et al., 1994), it is unlikely that inherited traits are all that account for differences in education duration among our participants. Thus, even in the case of the early visual cortex, experience over long time scales could enhance individual differences, disentangling the roles of phylogenetic and ontogenetic development on brain organization.

Our findings suggest a link between variability and plasticity in brain development. Not only was the visual RSFC more variable in the blind, but the variability was specifically increased in areas that showed reorganization because of blindness. Although this is a correlational finding, it seems plausible that the absence of an otherwise consistent experience would remove potential constraints on development, allowing more variability among individuals. This change might take place especially during brain development stages in which fine-tuning of cortical structure and anatomic connectivity is done. In other mammals, these include stages of pruning of exuberant connectivity, which is based in part on activity-dependent patterns (Innocenti and Price, 2005). Therefore, as suggested previously (Amedi et al., 2003; Sathian, 2005; Collignon et al., 2009), transient connectivity to the visual cortex (Dehay et al., 1984; Innocenti and Clarke, 1984; Dehay et al., 1988; Innocenti et al., 1988; Kennedy et al., 1989; Rockland and Van Hoesen, 1994; Rockland and Ojima, 2003; Innocenti and Price, 2005) that is typically pruned following visual experience may endure in blind humans to variable extents across individuals (thus not necessarily apparent in group-level analyses; Fine and Park, 2018). Changes to pruning as a result of visual or sensory experience was reported in other mammalian species (Nicolelis et al., 1991; Karlen et al., 2006; Henschke et al., 2018), and in nonhuman primates the absence of visual experience can also cause changes to corticogenesis (Magrou et al., 2017). Either mechanism could therefore introduce postnatal changes to connectivity. An alternative but nonexclusive account is that the variability reflected in the RSFC networks shown here stems from shorter-term changes in brain connectivity, such as those associated with unmasking of existing but dormant connections (Rauschecker, 1995; Hamilton and Pascual-Leone, 1998). Although a late-onset blindness group is needed to fully discern these two accounts, many studies have demonstrated that late-onset blindness is associated with lesser plasticity to early visual cortex compared with congenital blindness (Cohen et al., 1999; Burton et al., 2002a, b; Wittenberg et al., 2004; Fujii et al., 2009; Collignon et al., 2013; Carlo et al., 2020), suggesting that processes beyond unmasking are involved in generating nonvisual responses and RSFC in the congenitally blind. Regardless of the underlying mechanism, these data show that plasticity allows an increase in the breadth of potential outcomes for brain organization.

What are the sources of the differential variability between the blind and the sighted? In terms of visual experience, the blind participants are a homogenous group of congenitally and fully blind adults, without any ability to recognize visual shapes, virtually excluding different levels of visual experience as a basis for this variability. Although the origins of some of these differences may be genetically linked to the causes of blindness, it is worth noting that only some of the participants' blindness stemmed from clearly heritable conditions such as microphthalmia (Bardakjian and Schneider, 2011), and even in these cases the spatial profiles of connectivity did not seem to cluster based on blindness etiology (including for siblings; Fig. 3*A*), suggesting a relatively large effect of postnatal experiences. Instead, variability may be ascribed to two sources. The first is the absence of the typical visual input, which is characterized by specific and similar statistical properties (Simoncelli, 2003; Berkes et al., 2011). It is well known that visual experience influences brain organization and function (Wiesel and Hubel, 1963; Hubel and Wiesel, 1964; Sugita, 2004; Maurer et al., 2005; Ostrovsky et al., 2006; Sugita, 2008; Dehaene et al., 2010; Ruthazer and Aizenman, 2010; Espinosa and Stryker, 2012; Röder et al., 2013; Cloherty et al., 2016; Arcaro et al., 2017; Golarai et al., 2017; Gomez et al., 2019). As the visual system properties are evolutionarily tailored to the environment statistical properties (Simoncelli and Olshausen, 2001), confirmatory and typical external experience may strongly enforce typical organization and connectivity, that is, pruning the less-dominant and otherwise transient nonvisual inputs that may be more variable across individuals. A lack of a shared experience may lead to increased interindividual variability in the blind as (likely already variable) nondominant inputs may be strengthened by small environmental experiences, genetic predispositions, or random noise. This would lead to strengthening individual connectivity variance present already in neonates (Molloy and Saygin, 2021) and driving different individuals to strengthening connectivity with different systems.

Another (not mutually exclusive) source of variability could be individual adaptations to blindness, such as the compensatory use of other senses (Röder et al., 1999; Van Boven et al., 2000; Goldreich and Kanics, 2003; Collignon et al., 2009; Beaulieu-Lefebvre et al., 2011) and cognitive faculties (e.g., increased reliance and improved memory and verbal skills; Tillman and Bashaw, 1968; Pozar, 1982; Raz et al., 2007; Occelli et al., 2016; Dormal et al., 2016; Loiotile et al., 2019). Plasticity correlated to these different abilities has been found in the visual cortex of the blind (Amedi et al., 2003; Gougoux et al., 2005), and differential abilities and reliance on these modes of compensation (e.g., reading Braille books as opposed to listening to audiobooks) across individuals could lead to variability in visual system connectivity, as well as differential functional responses (van den Hurk et al., 2017; Abboud et al., 2019; Rosenke et al., 2020; Mattioni et al., 2020b). Here, we are unable to separate these two accounts completely. In a partial attempt to do so, we have shown here that the RSFC of the visual cortex to the left IFC is correlated to an individual's duration of formal education. However, most of the regions that showed changes in variability were not accounted for in this preliminary exploration. Furthermore, overall increased variability was not found in nonvisual sensory areas (auditory and somatosensory cortices), making it unlikely that experience or expertise in compensatory senses underlies the full variability. In fact, a cluster in the auditory cortex cortices showed decreased connectivity variability in blindness (similarly to a nonsignificant effect in the sensorimotor cortex; Fig. 2*E*), suggesting that the opposite effect, consistent reliance on audition in blindness, may also cause increased consistency of cross-modal connectivity. Future work should parse out the effects of specific environmental and personal factors affecting the postnatal reorganization in the blind.

Based on our exploratory clustering analysis, reorganization generates distinct spatial connectivity profiles. For example, connectivity between the visual and sensorimotor cortices varies between positive and negative values across individuals. This pattern suggests potentially informative changes in the link between the senses and the importance of reorganization regarding touch in different blind individuals. Most of the blind show connectivity between V1 and the IFC, but connectivity to the superior frontal cortex differs between subclades. Although a full characterization of individual profiles would benefit from additional correlates and an increased sample size, we can already gain two interesting insights. The first is that the most drastic form of reorganization associated with blindness, lateralized functional connectivity to the left frontal cortex (Liu et al., 2007; Yu et al., 2008; Wang et al., 2014; Burton et al., 2014; Qin et al., 2015; Striem-Amit et al., 2015), which has been described as allowing visual cortex functional recruitment for language (Bedny, 2017), is found only in a minority of the subjects (three of 25 participants; subclade VI; Fig. 3*A*). Overall, the RSFC between V1 and frontal cortex is quite variable (Figs. 1*B*, 2*E*,*F*) and more often bilateral (Figs. 2*D*, 3*A*). This observed heterogeneity of V1 connectivity can aid in resolving some of the current debate revolving the role of early visual cortex in blindness. The early visual cortex, at the group level, has shown recruitment in multiple tasks, including both low-level sensory processing and high-level cognitive functions (Sadato et al., 1996; Büchel et al., 1998; Weeks et al., 2000; Burton et al., 2002b; Amedi et al., 2003; Burton et al., 2004; Gougoux et al., 2005; Stilla et al., 2008; Bedny et al., 2011; Kanjlia et al., 2016; Mattioni et al., 2020a), challenging the definition of its functional role in blindness. This led to controversy about its capacity to plastically reorganize for nonvisual computations remote from its typical visual role (Bedny, 2017; Crollen et al., 2019; Seydell-Greenwald et al., 2020), as well as a debate on its place in the processing hierarchy (Amedi et al., 2003; Büchel, 2003; Watkins et al., 2012; Fine and Park, 2018). Beyond blindness, this debate has broader implications to the capacity for cortical plasticity also in other systems in congenital deafness (Lomber, 2017; Cardin et al., 2020) and handlessness (Hahamy et al., 2017; Striem-Amit et al., 2018a). Although our data cannot resolve this controversy, they offer an additional lens to inspect group-level data; it is possible that some of the contradictory group activations stem from different subgroups of blind participants (as seen in the ventral visual cortex; Rosenke et al., 2020) and that V1 in blindness may potentially assume different functional roles in different individuals. A similar approach may be further adopted to explain the variability found in functional recruitment profiles for the ventral visual cortex across individuals (van den Hurk et al., 2017; Rosenke et al., 2020; Mattioni et al., 2020b).

The spatial variability we report here can also interact with temporal variability. Recently, visual functional connectivity to the auditory cortex was shown to temporally vary more in blindness, as well as between task and rest (Pelland et al., 2017). This suggested that the visual cortex may not just take different roles across individuals as we propose here but may also vary its role and connectivity across time and tasks in a single blind person. Future studies will need to explore more deeply how individual differences manifest in blindness across different states and whether this information can aid in characterizing individual phenotypes (Greene et al., 2020). Different spatial RSFC patterns may reflect biases in engaging the visual cortex for longer durations in a specific functional network, even when no relevant task is attended, highlighting a more significant role for one function in each individual.

Importantly, studies in sighted individuals already show that individual differences can manifest across states (Gratton et al., 2018), allowing RSFC, even on its own, to be harnessed for predicting developmental outcomes (Kamps et al., 2020; Li et al., 2020; Whitfield-Gabrieli et al., 2020; Yu et al., 2021), clinical outcomes (Wisch et al., 2020; Prakash et al., 2021), and even therapeutic prescription (Fox and Greicius, 2010; Drysdale et al., 2017). Therefore, regardless of their sources, the existence of different reorganization profiles we observed may have clinical implications for vision rehabilitation. The causes of the high variability of outcomes of sight restoration attempts (Gregory and Wallace, 1963; Carlson et al., 1986; Ganesh et al., 2014; Huber et al., 2015) remain unknown, with some patients gaining little functional sight. As evident from cochlear implantation in deafness (Lee et al., 2001; Olds et al., 2016; Feng et al., 2018; cf. Lyness et al., 2013; Heimler et al., 2014; Land et al., 2016), variability in restoring a missing sense may depend on neural system retention as cross-modal reorganization may render it incapable of processing information of the original modality. Similarly, in visual restoration, some failed sight restoration attempts may have neural causes (Striem-Amit et al., 2011). In contrast to invasive methods that require an intact visual system, assistive and adaptive technologies such as sensory substitution devices are designed to use cross-modal translations. For example, sensory substitution devices that convert visual images into sounds or touch (Bach-Y-Rita et al., 1969; Meijer, 1992; Capelle et al., 1998; Striem-Amit et al., 2012) could benefit from cross-modal plasticity of specific senses (Brown et al., 2011; Arnold et al., 2017). In late-onset vision loss because of age-related diseases (e.g., macular degeneration, glaucoma, cataracts) there is a dizzying selection of sensory aids and substitution techniques. For the task of reading alone, approaches include refreshable Braille displays, screen readers, and optical and electronic aids using touch, audition, and vision, respectively. Similar diversity exists for navigation needs (guide dog, white cane, electronic canes, smart glasses). Matching technologies that are most effective based on the individual neural plasticity profile may aid in individually tailored, personalized medicine and assistive technology in sight rehabilitation of visual disorders.

In conclusion, we showed that in the absence of sensory experience because of blindness, brain reorganization generates larger interindividual variability beyond the individual differences found in the typical sighted population. Variability is increased especially for areas that have reorganized in their connectivity to V1 because of blindness, and blind individuals show different spatial patterns of connectivity of their visual cortex. This finding suggests an important role for experience in determining the individual variability of neural organization. Additionally, these results highlight the need to consider idiosyncratic profiles of plasticity in tailoring rehabilitation plans for individuals with sensory deficits.
