# Supplementary figures and images for: The Role of Visual Experience in Individual Differences of Brain Connectivity
Source: J Neurosci. 2022 Jun 22;42(25):5070–84. doi: 10.1523/JNEUROSCI.1700-21.2022 (PMC9233442; doi:10.1523/JNEUROSCI.1700-21.2022)

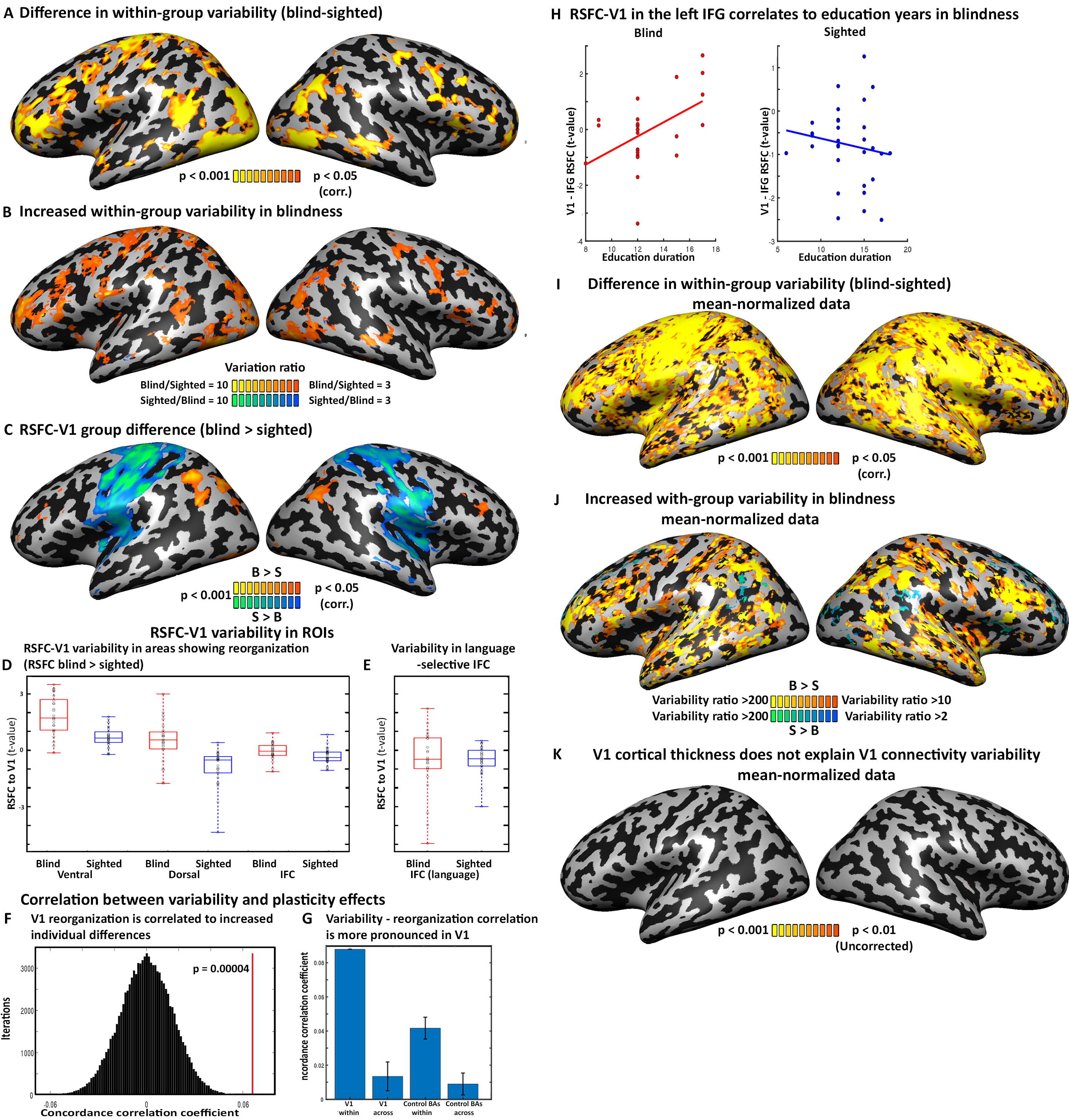

Supplement: Figure 1-1 — Reproducing main analyses with global signal regression as a preprocessing step. Main analyses were reproduced when including global signal regression as a preprocessing step. A, Replicating Figure 1A, the difference in within-group variability between the groups is significant in various parts of the brain, including in the frontal lobe. B, Replicating Figure 1B, directional comparison of the within-group variability difference (ratio of blind intragroup variability divided by sighted intragroup variability >3) shows that the blind have increased variability in most of the regions differing in their variation between the groups. This suggests a stabilizing effect of visual experience on visual cortex developmental functional connectivity. C, Replicating Figure 2D, increased V1-seeded RSFC in blindness is found in the visual streams as well as in the bilateral IFC. Global signal regression is known to introduce anticorrelation, distortion of group differences, and exacerbation of distance-dependent motion artifacts (Murphy et al., 2009; Anderson et al., 2011; Power et al., 2012; Saad et al., 2012; Satterthwaite et al., 2012; Gotts et al., 2013; Hahamy et al., 2014; Ciric et al., 2017), thus this somewhat quantitatively differs from Figure 2D for group differences distant from the seed ROI. D, E, Replicating Figure 2, E and F. The blind show increased variability in their V1-seeded RSFC to left ventral and dorsal streams and IFC frontal areas as well as decreased connectivity to V1 in the sensorimotor cortex. E, Within the areas showing increased RSFC in the blind (Extended Data Fig. 1-1C). F, Sampled in a language-selective IFC ROI, defined by preference toward words compared with pseudowords. Box plots are presented for the blind and sighted in red and blue, respectively. The central mark indicates the median, and the bottom and top edges of the box indicate the 25th and 75th percentiles, respectively. Individual participant data are presented in circles. F, Replic [file ns-JN-RM-1700-21-s01.tif]

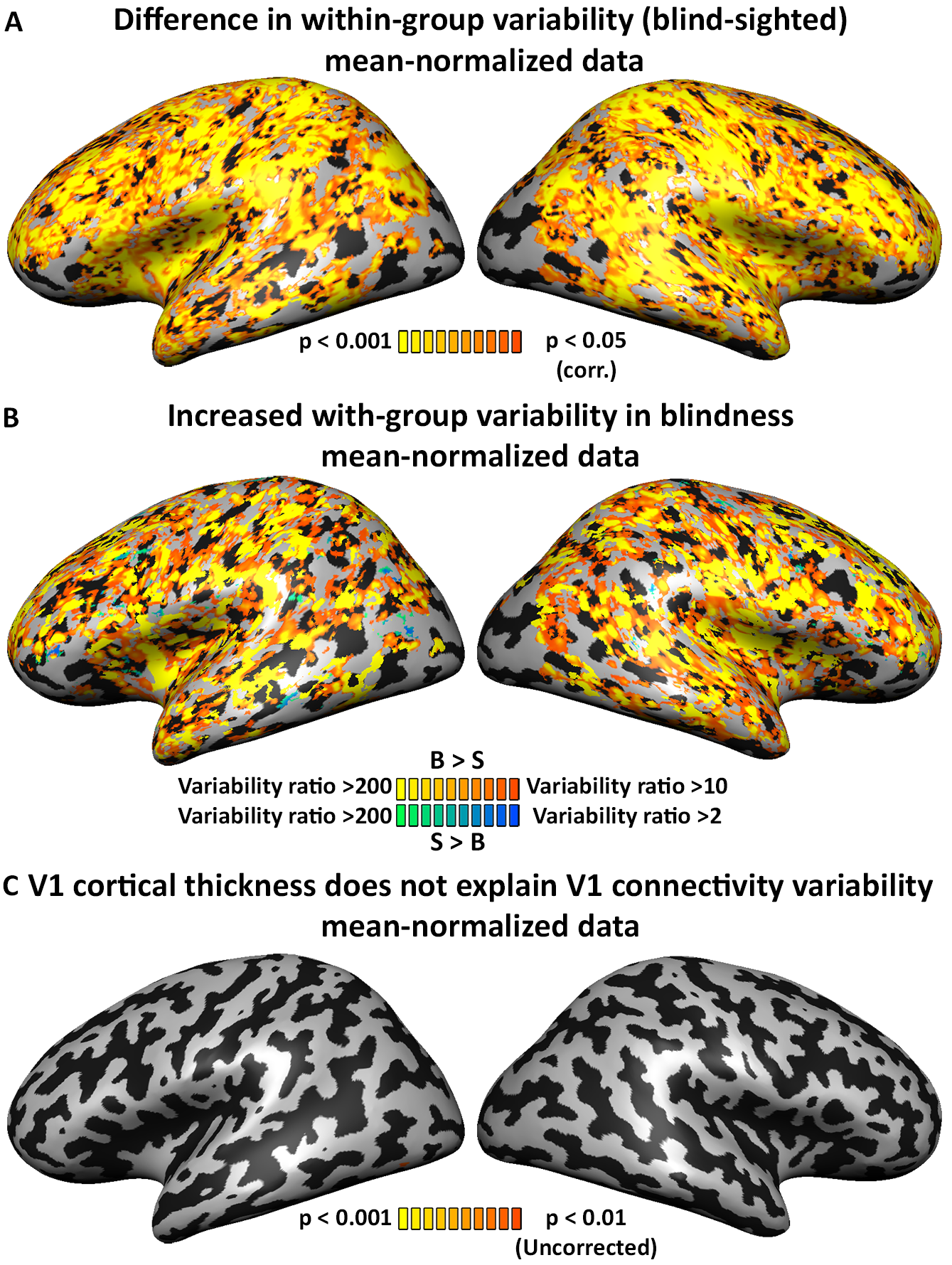

Supplement: Figure 1-2 — Variability increase in blindness is found regardless of changes to mean RSFC. A, The difference in variability between the groups (replication of the analysis shown in Fig. 1A when demeaning the V1-RSFC maps) is significant in various parts of the brain, including in the frontal lobe, even when controlling for the higher mean RSFC values in the blind. This difference in variability is even more robust, compared with Figure 1A, and covers larger portions of cortex. B, The blind show increased variability (ratio of blind intragroup variability divided by sighted intragroup variability >10; replication of the analysis shown in Figure 1B when demeaning the V1-RSFC maps) in the vast majority of the regions differing in their variation between the groups when controlling for the higher mean RSFC values in the blind. This supports the conclusion that individual differences increase in blindness. C, V1 cortical thickness, showing a difference between the groups, is not correlated to the functional connectivity from V1 in the blind (or across both groups, also showing no significant effect), even at a lenient threshold of p < 0.01 uncorrected, and can therefore not account for the change in connectivity variability. Download Figure 1-2, TIF file. [file ns-JN-RM-1700-21-s02.tif]

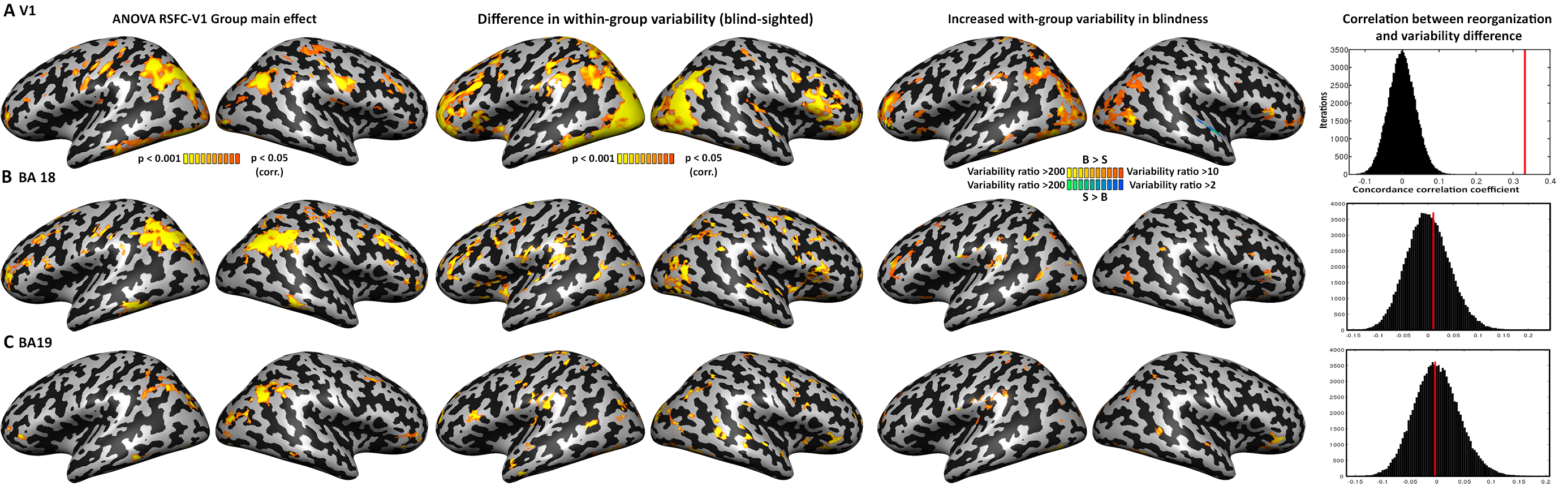

Supplement: Figure 1-3 — Variability changes in functional connectivity to association visual cortex. A–C, Analyses of variability are presented for visual areas V1 (A, replicating analyses in the article), Brodmann area 18 (B), and Brodmann area 19 (C). Left column, Analyses of the difference in within-group variability between the blind and sighted (replicating Fig. 1A for V1) show changes to variability because of blindness are not limited to V1. Second column, A main effect of sight across the cohorts is depicted. (replicating Fig. 2A for V1). Third column, Directional comparison of the within-group variability difference (ratio of blind intragroup variability divided by sighted intragroup variability >3) shows that the blind have increased variability in most of the regions differing in their variation between the groups (replicating Fig. 1B for V1). Far right, The concordance correlation coefficient was calculated between the RSFC group difference and RSFC change in variability for the seeds (red line) and compared with a spatial permutation test (distribution in black). Only V1 shows correlation between the two effects (replicating Fig. 2G), whereas BA18 and BA19 do not (BA18 CCC = 0.01, p = 0.81, BA19 CCC = −0.003, p = 0.94). Download Figure 1-3, TIF file. [file ns-JN-RM-1700-21-s03.tif]

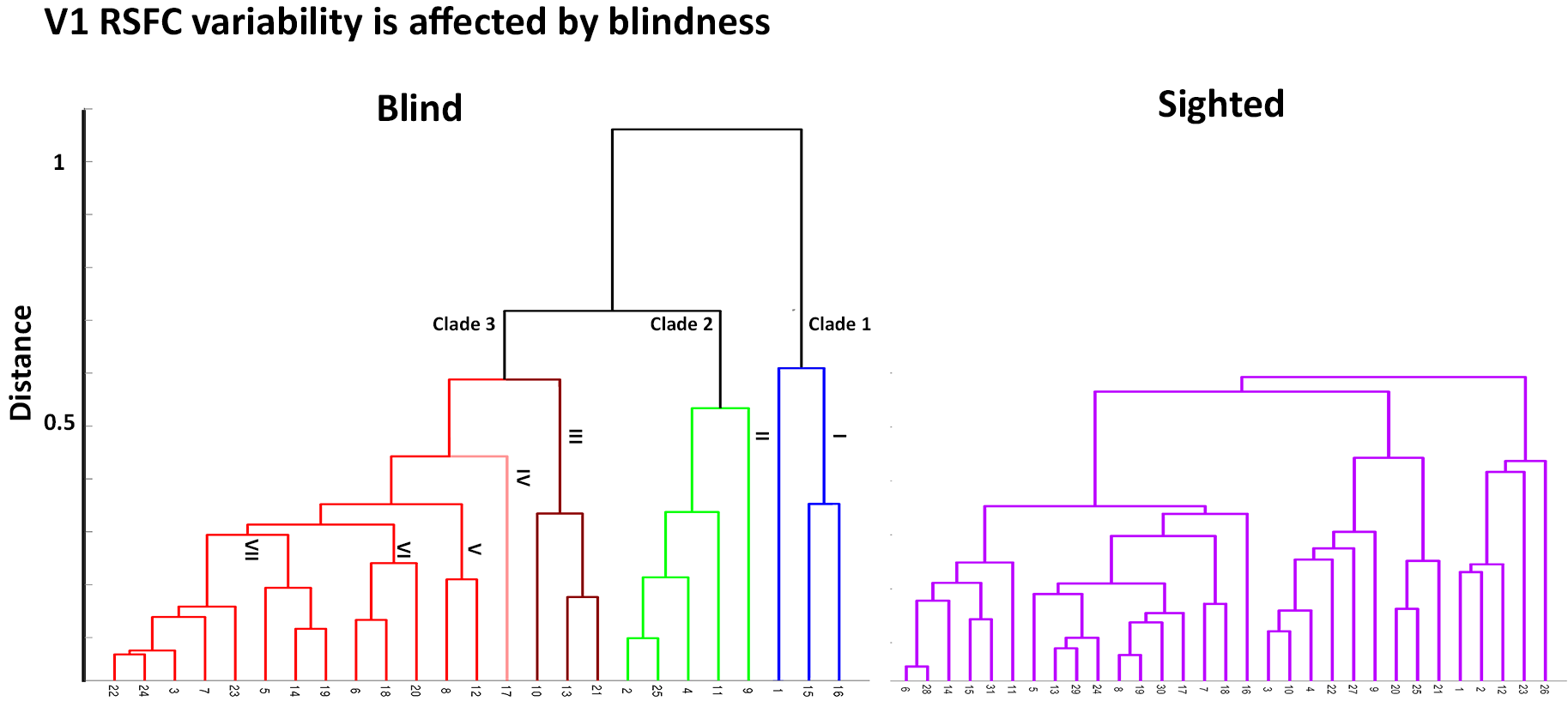

Supplement: Figure 3-1 — V1 RSFC variability is affected by blindness. V1-RSFC of each individual participant to each Brodmann area was used to compute hierarchical clustering of RSFC patterns within each group. Left, Replication of Figure 3A showing the clustering of the blind into three main clades and subclades. Right, The comparable clustering dendrogram in the sighted group. Although division to a similar number of clades and subclades can be found, the overall distances were lower, as evident by the y-axis, and the distances were significantly smaller than in the blind group (t(52) = 3.17, p = 0.007). Download Figure 3-1, TIF file. [file ns-JN-RM-1700-21-s04.tif]
